# Established Models and New Paradigms for Hypoxia-Driven Cancer-Associated Bone Disease

**DOI:** 10.1007/s00223-017-0352-6

**Published:** 2017-11-02

**Authors:** Thomas R. Cox, Janine T. Erler, Robin M. H. Rumney

**Affiliations:** 1 The Garvan Institute of Medical Research and The Kinghorn Cancer Centre, Cancer Division, St Vincent’s Clinical School, Faculty of Medicine, UNSW Sydney, Sydney, NSW 2010 Australia; 2 Biotech Research & Innovation Centre (BRIC), University of Copenhagen (UCPH), Ole Maaløes Vej 5, 2200 Copenhagen, Denmark; 3 Institute of Biomedical and Biomolecular Sciences, School of Pharmacy and Biomedical Sciences, University of Portsmouth, Portsmouth, PO1 2DT UK

**Keywords:** Cancer, Hypoxia, Bone, Metastasis, Lysyl oxidase

## Abstract

The five-year survival rate for primary bone cancers is ~ 70% while almost all cases of secondary metastatic bone cancer are terminal. Hypoxia, the deficiency of oxygen which occurs as the rate of tumour growth exceeds the supply of vascularisation, is a key promoter of tumour progression. Hypoxia-driven effects in the primary tumour are wide ranging including changes in gene expression, dysregulation of signalling pathways, resistance to chemotherapy, neovascularisation, increased tumour cell proliferation and migration. Paget’s seed and soil theory states that for a metastasising tumour cell ‘the seed’ it requires the correct microenvironment ‘soil’ to colonise. Why and how metastasising tumour cells colonise the bone is a complex and intriguing problem. However, once present tumour cells are able to disrupt bone homeostasis through increasing osteoclast activity and downregulating osteoblast function. Osteoclast resorption releases growth factors from the bone matrix that subsequently contribute to the proliferation of invasive tumour cells creating the vicious cycle of bone loss and metastatic cancer progression. Recently, we have shown that hypoxia increases expression and release of lysyl oxidase (LOX) from primary mammary tumours, which in turn disrupts bone homeostasis to favour osteolytic degradation to create pre-metastatic niches in the bone microenvironment. We also demonstrated how treatment with bisphosphonates could block this cancer-induced bone remodelling and reduce secondary bone metastases. This review describes the roles of hypoxia in primary tumour progression to metastasis, with a focus on key signalling pathways and treatment options to reduce patient morbidity and increase survival.

## An Introduction to Hypoxia

Hypoxia is the deficiency of oxygen that commonly occurs within tumours. There are two broad classes of hypoxia, transient hypoxia which is associated with inadequate blood flow, while chronic hypoxia is the consequence of the increased oxygen diffusion distance due the rate of tumour growth exceeding the rate of neovascularisation [1]. Both types of hypoxia are clinically correlated with poor outcome for patients. The presence of hypoxia and the lack of vascularisation represent a double-edged sword of stimuli for cancer progression and obstacles against cancer treatment. Hypoxia triggers changes in gene expression that contribute to tumour progression via several mechanisms including neovascularisation and increases in cell proliferation, motility, and migration leading to metastasis [2–4]. Treatment of hypoxic cancers is impeded in several ways including (i) the lack of functional vasculature reduces access of chemotherapeutic drugs to cancers via the circulatory system; (ii) changes in acidity as a result of hypoxia alter the stability and activity of chemotherapeutic agents; (iii) some chemotherapeutic drugs require oxygen to generate free radicals that contribute to their cytotoxicity; (iv) hypoxia typically reduces the rate of proliferation of cancer cells, the very mechanism which chemotherapy targets; and (v) through hypoxia-induced chemoresistance within cancer cells mediated through adaptation by post-translational and transcriptional changes [5].

## Epidemiology and Roles of Hypoxia in Primary Bone Cancers

Hypoxia decreases survival rates in all primary bone cancers. The most common is osteosarcoma which arises from primitive bone forming mesenchymal cells and accounts for 56% of all cases of primary bone cancer [6]. For osteosarcoma 5-year survival rates are age related with ~ 60% survival for patients under 19 years of age [6] and just 30% for those aged 50 and over [7]. Immunohistochemical labelling for the hypoxia marker HIF-1α in clinically derived osteosarcoma tumour samples correlates with pathologic stage, and tumours from patients with medium to strong HIF-1α expression had decreases in both overall survival and disease-free survival [8].

Ewing sarcoma is the second most common paediatric primary bone cancer after osteosarcoma, accounting for 34% of malignant bone tumours with a 58% five-year survival rate for children and adolescents [6]. One study has suggested that ES patients aged 26 and above are in the highest risk group, since response to chemotherapy was only observed in 50% of patients with localised disease, and just 9% of patients with metastatic disease. The five-year survival rate in this group is only 37% [9]. In Ewing sarcoma, hypoxia-induced expression of HIF-1α has been shown in primary ES tumours [10] and co-localises with the programmed cell death-associated protein caspase 3 [11].

Chondrosarcoma is a neoplastic cartilage tumour that is rare in children but more common in adults making it the third most common paediatric primary bone cancer but the second most common in adults [6]. Five-year survival rates vary tremendously with progression of chondrosarcoma from 93% in juxtacortical chondrosarcoma to 0% in dedifferentiated chondrosarcoma where most patients die within a year of diagnosis [12]. In clinical samples of high-grade chondrosarcoma, elevated levels of HIF-1α inducible matrix metalloprotease 2 and galectin 1 have been detected, consistent with increased hypoxia signalling [13, 14].

In primary cancers, hypoxia is often the key driver of progression to the point where metastasis to other organs occur. This is particularly important in multiple myeloma, a cancer of the plasma cells in bone marrow which spreads to the calcified portion of the bone, and primary cancers of breast and prostate which are also highly bone tropic leading to secondary bone cancers.

## Pathways and Mechanisms of Hypoxia-Induced Cancer Progression in Primary Bone Cancers

### HIF-1α–VEGF–CXCR4 Pathway

The most important mediators of the cellular response to hypoxia are the transcription factor hypoxia inducible factors 1 and 2 (HIF-1 and HIF-2) [15]. The best studied is HIF-1, a heterodimer of the HIF1α and HIF1β two subunits. The functionality of HIF-1 as it drives the response of the tumour to hypoxia is determined by the availability of HIF1α, which is up-regulated under hypoxia. HIF1α is an upstream regulator of vascular endothelial growth factor (VEGF), a signal protein that promotes angiogenesis by acting upon the VEGF receptors VEGFR1 and VEGFR2 (the latter also known as kinase insert domain receptor) [16]. HIF1-α and VEGF drive expression of C-X-C chemokine receptor type 4 (CXCR4), a chemokine receptor for stromal cell-derive factor-1 (SDF-1) α (also known as CXCL12). There is evidence that HIF1α directly induces VEGF and CXCR4 transcription by binding to hypoxia response element regions [17], while VEGF has also been implicated in CXCR4 upregulation of angiogenic vessels in glioblastoma [18]. As such, it has been shown that the HIF-1α–VEGF–CXCR4 axis is a key driver in primary bone cancers and many of its effects can be prevented with the CXCR4 inhibitor AMD3100 [19, 20].

HIF-1α is responsible for multiple effects that have been associated with enhanced cancer progression and poorer patient outcome. These include enhanced tumour cell proliferation [21, 22], migration [21, 23, 24], and metastasis [10, 23, 25]. In primary osteosarcoma, HIF-1α expression in tumour biopsies is seen to be significantly correlated with metastasis, and in vitro data reveal that VEGF expression driven by HIF-1α is associated with cell migration [26]. In Ewing sarcoma cells, siRNA interference of HIF1-α in vitro reduces the hypoxic induction of VEGF [11]. Clinically, serum VEGF is elevated in patients with ES, which correlates strongly with expression of HIF1-α in the primary tumour [27]. In ES, tumour cells that line blood lakes were shown to express HIF-1α which co-localised with the hypoxia marker pimonidazole in conjunction with vasculogenic mimicry [28]. In ES cells, siRNA interference of HIF1-α in vitro also reduces hypoxic induction of VEGF [11]. Clinically, serum VEGF is elevated in patients with ES, which correlates strongly with expression of HIF1-α in the primary tumour [27]. Although normal healthy cartilage is typically a hypoxic avascular tissue supplied only via diffusion, chondrosarcoma is vascularised. This is likely the result of the elevated HIF-1α, observed in chondrosarcoma biopsies, and is required for hypoxia-induced VEGF expression in chondrosarcoma cell lines [29]. Hypoxia selectively augments CXCR4 expression through HIF-1 activation in several cell types including cancer cells [30]. Hypoxia increases CXCR4 expression in both ES and chondrosarcoma cell lines grown in vitro or as tumours in vivo in nude mice [31, 32]. This was associated with increased VEGF secretion in vitro detectable by ELISA and the functional endothelial cell tubule formation assay, a proxy for vascularisation [32]. The subsequent ES cell migration (which can be inhibited by AMD3100) is driven towards the CXCR4 ligand stromal cell-derived factor (SDF1)-α which is commonly found in sites of metastasis in vivo [31].

In osteosarcoma, cell invasion across transwells in vitro is increased under hypoxic conditions reliant upon HIF-1α promoting VEGF-A expression [26]. This could be decreased by inhibition of HIF-1α, or treatment with the anti-CXCR4 drug AMD3100 [26, 33]. Of note, hypoxia-treated osteosarcoma cells injected into the tail vein of mice led to increased lung metastases compared to normoxic controls, an effect that was ablated if cells were also pre-treated with AMD3100 [33]. Finally, in a mouse model of chondrosarcoma, siRNA inhibition of CXCR4 decreased overall tumour volume and incidence of metastases to the lungs [32]. Overall, expression of CXCR4 is a poor prognostic indicator that is associated with an increase in many of the processes that are associated with cancer progression including cell migration and metastasis.

HIF-1α is also up-regulated in breast and prostate cancers [34]. In breast cancers with stromal fibroblasts that secrete SDF-1 and CXCL-12, the tumours exhibit increased growth, angiogenesis and invasion [35]. Some of the response of breast cancer to CXCR4 may be determined by the presence of estrogen receptors, as the CXCl-12 signalling axis is important for the estrogen-mediated growth of breast cancer cells [36]. The presence of CXCR4 has also been shown to correlate with metastatic disease in prostate cancer, where xenograft tumours that over expressed CXCR4 grafted into immunodeficient NOD/SCID mice exhibited increased growth, vessel density and metastasis [37]. As in primary bone tumours, expression of CXCR4 causes cancer progression, and, in the case of breast or prostate cancer, may increase the risk of metastasis to bone.

## The Neuropeptide Y-Dipeptidyl Peptidase-IV Axis

There are certain signalling pathways that are specific to individual types of cancer. In ES, the main underlying cause is chromosomal translocation resulting in the production of fusion oncoprotein. In ~ 90% of all cases, this involves the fusion of the amino-terminal of the Ewing sarcoma breakpoint region 1 gene (EWSR1) with the DNA-binding domain of the *ETS* gene family member *FLI1* to generate the EWS-FLI1 fusion protein [38, 39]. Neuropeptide Y (NPY) is a transcriptional target of the fusion oncoprotein EWS-FLI1. Elevated release of NPY by Ewing sarcoma tumours is associated with site-specific metastasis including thoracic extra-osseous, bone and brain. Use of short hairpin RNA (shRNA) to reduce NPY expression in an Ewing sarcoma cell line then injected into mice exhibited, reduced bone destruction as quantified by µCT [38]. While shRNA may not be readily translatable to the clinic, selective radiolabelled peptides have recently been investigated as potential NPY therapies [40]. Such radiolabelled peptides have been used clinically for nearly three decades for a range of treatments, the first being an analogue for the growth hormone-inhibiting molecule somatostatin. In cancer, radiolabelled peptides are also used to bind specific membrane-associated receptors enabling targeted delivery of molecules for the detection and treatment [41]. Examples of this include, a radiolabelled Neuropeptide Y analogue which can be internalised by neuroblastoma cells [42], a radiolabelled luteinizing hormone releasing hormone which selectively binds to ovarian cancer cells in vivo to enhance cell apoptosis [43], and the somatostatin analogue octreotide (Sandostatin) for treatment of neuroendocrine tumours [44]. However, to our knowledge, no radiolabelled peptides have been used to target Ewing sarcoma.

Under normoxic conditions, NPY acts upon the Y1R and Y5R receptors to stimulate cell death in Ewing sarcoma; however, hypoxia alters the expression of NPY receptors by inducing Y2R expression instead. Hypoxia also increases the expression of the enzyme dipeptidyl peptidase IV (DPP-IV) which truncates NPY. The resulting NPY3-36 does not bind Y1R to induce cell death but acts as selective agonist on Y2R. Hypoxia also increases expression of Y2R on endothelial cells which are sensitive to NPY3-36 to drive angiogenesis. Furthermore, DPP-IV drives proliferation and migration of ES cells [45]. A clinical study by the same group recently found that NPY was systemically increased in Ewing sarcoma patients and was associated with metastasis [46]. Although direct clinical targeting of DPP-IV has not been investigated in Ewing sarcoma, it has been widely investigated in other diseases where DPP-IV is known to be a contributing factor. To date, there are several inhibitors of DPP-IV that are being given to diabetes patients [47], yet to the best of our knowledge there are no reports available of DPP-IV inhibitors being used to treat Ewing sarcoma.

## Targeting Hypoxia in Primary Tumours

One way to turn the tables on hypoxia-driven chemoresistance is to develop therapies that rely on, or directly target hypoxia-driven effects or the hypoxic microenvironment. One such approach utilises bioreductive or hypoxia-activated pro-drugs (HAPs), where the central premise is that inactive pro-drugs are delivered to the hypoxic regions of the tumour whereupon they are enzymatically activated, or spontaneously reduced to release their cytotoxic payloads that target cancer cells. This approach works best where hypoxia within the tumour of the patient is seen to be responsible for the failure of standard-of-care treatment [48].

There are several HAPs that have either recently or are currently undergoing clinical trials including AQ4N, PR-104, and TH-302. AQ4N becomes AQ4 after activation in hypoxic conditions whereupon it non-covalently binds to DNA to facilitate anti-tumour activity and selectively inhibits the activity of the DNA separation enzyme topoisomerase II [49]. Mice injected with LNCaP prostate tumour cells and treated with AQ4N in conjunction with the anti-androgenic drug bicalutamide exhibited delayed tumour growth which was significantly greater than bicalutamide alone [50]. AQ4N has been delivered to human breast tumours as part of a phase I clinical trial [51] and shown promise. However to date, there are no studies available where AQ4N has been used in primary bone cancers either in vitro or in vivo.

PR-104 is a phosphate ester that is hydrolysed and then reduced under hypoxic conditions to release amine and hydroxyl-amine nitrogen mustards that selectively cross-link DNA in hypoxic cells [52]. PR-104 has been given to patients with acute myeloid and acute lymphoblastic leukaemia in phase I/II trials whereupon measurable anti-leukaemic responses were observed at doses of 3 g/m^2^ and above [52].

TH-302 (evofosfamide) is a nitroazole-containing pro-drug that selectively releases bromo-isophosphoramide which cross-links DNA [53, 54]. In the clinic, TH-302 has been tested in phase I trials against relapsed/refractory leukaemia where it had little effect [55]. Although phase II trials of TH-302 together with the chemotherapy drug doxorubicin in patients with soft tissue sarcoma have suggested a favourable response, in line with other first line chemotherapies [56]. However, a recent phase III trial found that TH-302 with doxorubicin did not increase survival compared to doxorubicin alone [57]. Other phase III clinical trials of TH-302 in pancreatic cancer found no significant increase in 1-year survival rate with TH-302 and although there was a small yet significant increase in progression-free survival this study did not reach primary overall survival endpoints [58, 59]. These data suggest that while TH-302 may have some limited effect on survival, it varies depending upon the specific form of cancer. While clinical trial data are not yet available to assess the potential of pro-drugs such as TH-302 on primary bone cancers, the effects of TH-302 have been studied by in vitro and in vivo methods. Bone destruction around the tumour site created by osteosarcoma cells after injection into the tibial marrow cavity of nude mice is reduced by TH-302 administration, which also reduces the incidence of metastases to lung [60] which at least suggests a mechanism by which TH-302 might work if applied to human disease. The potential role for TH-302 in combatting chondrosarcoma at a very early stage has been highlighted in one study which showed that hypoxic regions of in vitro chondrosarcoma cell spheroids exhibit a cytotoxic response to TH-302 [61]. Furthermore, TH-302 triggers cell cycle arrest and apoptosis in multiple myeloma cells under hypoxic conditions, while in vivo treatment of the 5T33MM mouse model of multiple myeloma with TH-302 triggered apoptosis of multiple myeloma cells within the bone microenvironment [62]. Adding to this body of evidence, mice transplanted with osteolytic MDA‐MB‐231‐TXSA, the treatment with TH-302 was found to be selectively cytotoxic under hypoxic conditions and able to suppress tumour growth [63]. In another in vivo study, TH-302 worked with doxorubicin treatment to enhance anticancer effects in hypoxic regions, but it was found to increase toxicity mirroring that seen in clinical trials for this combination in soft tissue sarcoma [64]. TH-302 has also been tested in combination with the chemotherapeutic drug docetaxel against prostate cancer in a murine xenograft model with the PC-3 cell line, whereupon it had no additional effect to docetaxel alone. However, a separate study reported that TH-302 in combination with docetaxel had an enhanced growth delay effect on tumours in athymic nude mice with PC-3 cells [65]. The fact these two separate studies both investigating the effect of TH-302 upon mice injected with PC-3 prostate cancer cells can show such different results strongly suggests that the working requirements for TH-302 may be specific not only to the type of cancer under investigation, but also to the specific combination of other chemotherapeutic agents used, the strain of mouse model and other holistic considerations. These studies suggest that in conjunction with the correct combination of chemotherapeutic drugs, TH-302 might be useful, not only for targeting hypoxic primary bone tumours, but also cancers that have a tendency to spread to bone after metastasis.

Another ‘hypoxia targeting’ approach entails the direct targeting of HIF at the mRNA and protein levels. There are a number of micro RNAs that act as inhibitors of HIF-1α in healthy tissues but are downregulated in cancers allowing for an increase in HIF-1α expression such as miR-33b which is downregulated in osteosarcoma [21] and oncosuppressive in breast cancer tissues [66]. Overexpression of miR-33b in osteosarcoma cell lines reduces HIF-1α together with cellular proliferation and migration [21]. Although it is intriguing to note that targeting a specific microRNA under in vitro conditions can be used to modulate HIF-1α expression in cell lines, this is still a long way from any clinical application. RNA is an inherently unstable molecule so delivery of RNA in such a way that a patient may benefit from it is a huge challenge. Lung tissue has been a particular target for RNA delivery as the RNA may be deliverable via inhaler [67] but delivery to tumours that are not located within the lung poses a greater challenge. There is at least one ongoing phase I clinical trial targeting multiple solid tumours (including multiple myeloma) with a mimic for the apoptosis regulator miR-34 delivered via lipid nanoparticles [68]. Although potentially very exciting, at the time of writing the results of this trial (NCT01829971) are yet to be reported. The function of HIF-1 can also be interrupted at the protein level. Acriflavine, a drug which is more widely used as an antiseptic, binds directly to HIF-1α protein to prevent dimerization with HIF-1β thereby preventing the formation of functional HIF-1 heterodimer. This approach has been used to drive an arrest in the growth of PC-3 prostate cancer xenografts in severe combined immune deficiency (SCID) mice [69]. More recently, Acriflavine has been demonstrated to enhance the anti-tumour activity of the tyrosine kinase inhibitor Sunitinib in the syngeneic 4T1 breast cancer cell and BALB/c mouse model [70]. Overall, these studies demonstrate that multipronged approaches for targeting the overexpression of HIF-1 in response to hypoxia may yield promising results.

## Secondary Bone Cancers

All secondary metastatic bone cancers begin as primary cancers elsewhere in the body. The primary cancers, typically triggered by hypoxia, undergo progression to metastasis at which point the cells of bone-tropic cancers can invade the skeletal system. It has been well over a century since Stephen Paget’s visionary seed and soil hypothesis of 1889, when he wrote “When a plant goes to seed, its seeds are carried in all directions; but they can only live and grow if they fall on congenial soil” [71]. While this incisive analogy has stood the test of time in many ways, it is an over simplification to suggest that metastatic cancers simply “fall” into the bone, instead it may be more accurate to state that bone-tropic cancers actively home into bone [72].

Once metastatic cancer cells colonise the bone microenvironment, they initiate a dysregulation of bone homeostasis typically referred to as the ‘vicious cycle’ [73]. Under normal conditions, the bone forming activities of osteoblasts and resorbing activities of osteoclasts are coupled. Localised autocrine and paracrine intercellular signalling together with systemic factors regulate the co-ordinated activities of osteoblasts and osteoclasts to maintain healthy bone metabolism [74]. This tightly regulated system becomes disrupted by the arrival of metastatic cancer cells to the bone microenvironment, which secrete osteoclast stimulating factors that drive increased osteoclastic activity and osteolysis. As a result of increased bone turnover, there is an increase in the release of sequestered bone-derived growth factors that subsequently promote growth of the metastatic cancer cells. The result of the vicious cycle is net bone loss in osteolytic lesions that is associated with hypercalcemia, increased risk of bone fracture and intense neuropathic pain [75]. Some of these effects can be controlled at least temporarily by treatment with anti-resorptive bisphosphonate drugs that bind calcium in the bone and trigger apoptosis when resorbed by osteoclasts [76]. Another approach is to target receptor activator of nuclear factor-κβ (RANK) ligand (RANKL), the main protein required for osteoclast differentiation, which can be blocked with the monoclonal antibody denosumab [77].

In the 128 years since Paget’s hypothesis much has been learnt about how cancers spread to bone, yet despite this length of time and research, secondary metastatic bone cancer remains a terminal condition. In the following sections, this review will discuss what is known about some of the most important secondary metastatic cancers of the bone, and how revelatory paradigm shifts in our understanding of secondary bone cancers could help to improve future outcomes.

## Multiple Myeloma

Multiple myeloma (MM) is a cancer of the plasma cells located within the bone marrow which metastasises to the cortical and trabecular bone. The first study to investigate the interaction between bone marrow hypoxia and MM utilised the 5T2MM myeloma mouse model to demonstrate that although healthy and myelomatous bone marrow is hypoxic, the environment is more hypoxic in myelomatous bone marrow. These differences in hypoxia were mirrored by HIF1-α expression levels in the myelomatous bone marrow. Expression of HIF1-α was highest across all populations of 5T2MM cells in the pre-angiogenic stage, suggesting that microvessel density is increased in myelomatous bone marrow to decrease hypoxia concomitantly with increased HIF-1α expression. Under in vitro hypoxic conditions, CD45^−^ MM cells underwent apoptosis while tumour infiltrating CD45^+^ MM did not, suggesting that hypoxia is most important in the early stages of MM progression [78]. The role of HIF-1α in MM was expanded upon in a subsequent study that identified high constitutive expression of HIF-1α in MM cells. Under healthy conditions HIF-1α inhibits c-Myc activity, but in MM deregulated c-Myc becomes oncogenic and facilitates HIF-1α induced VEGF production and secretion to increase angiogenesis [79]. Indeed, in clinical biopsies, HIF-1α and VEGF receptor expression (in the phosphorylated active form of VEGFR2/KDR) were up-regulated in 40% of samples and were associated with increased angiogenesis [80]. Intuitively, suppression of HIF-1α in MM cells downregulates pro-angiogenic genes including VEGF and subsequently reduces tumour burden in both subcutaneous and intratibial mouse models of MM. This was shown to be by reducing VEGF expression and vessel number within tumours which had the additional beneficial effect of reducing bone destruction [81]. In addition to increased angiogenesis, hypoxia can promote epithelial to mesenchymal transition of MM cells. This results in a decreased expression of E-cadherin, which is thought to make MM cells less adhesive, and allow them to invade and enter into the blood stream and travel to new sites to form secondary metastasis in organs such as the bone. MM cells are chemotactically attracted to sites of hypoxia-induced CXCR4 expression, an effect that can be blocked by AMD3100 [82]. As with many other cancers, there is a switch between expression of E-cadherin and N-cadherin during the epithelial–mesenchymal transition (EMT) of MM. Expression of E-cadherin is decreased in response to hypoxia [82]. N-cadherin expression has been associated with increased integrin-α8 expression where increased N-cadherin has been observed in LP1 and RPMI-8226 MM cells and coincides with elevated expression of integrin-α8. Thus, it is thought to drive expression of genes associated with stemness including HIF-1α [83]. Clinically, N-cadherin expression is found in malignant plasma cells from a subset of MM patients, and injection of labelled MM cells into mice has revealed that increased expression of N-cadherin helps to retain MM cells in the bone marrow. Finally, in vitro co-cultures of N-cadherin expressing MM cells with pre-osteoblastic cells acts to inhibit osteoblastogenesis [84]. Thus, hypoxia can be seen to increase angiogenesis via multiple pathways, to drive EMT of MM, and chemotaxis of metastatic MM cells to the mineralised bone. Intriguingly, an in vitro study on hypoxia-resistant MM cells found that these cells secreted an increased number of exosomes compared to parent cells. In these, exosomal micro RNA 135b (miR-135b) indirectly increased HIF-1α in endothelial cells through suppression of Factor Inhibiting HIF-1α, thereby increasing endothelial tubule formation as a model for angiogenesis [85].

## Breast Cancer

One pathway in breast cancer that occurs downstream of hypoxia and HIF-1α involves transforming growth factor (TGF)-β signalling. Hypoxia drives HIF-1α signalling in parallel with TGF-β in the estrogen receptor negative (ER^−^) human cell line MDA-MB-231 under in vitro and in in vivo conditions. Hypoxia induces HIF-1α expression in human MDA-MB-231 cells in vitro and at sites of bone metastasis in vivo. Knockdown of HIF-1α reduces formation of osteolytic bone lesions in mice and decreases staining for the endothelial cell marker CD31, a marker for angiogenesis in sites of bone metastasis. HIF-1α knockdown or TGF-β blockade has also been shown to reduce bone metastasis and increase survival in mice implanted orthotopically with human MDA-MB-231 breast cancer cells [86]. The interaction between HIF and TGF-β may be mediated by microRNAs, since HIF induces miR-191, which in turn promotes TGF-β signalling. miR-191 also promotes proliferation, migration and invasion of human MCF7 breast cancer cells, while protecting against radiation induced cell death, suggesting a mechanism for hypoxia-induced resistance to radiotherapy [87].

Notch signalling is also an important signalling pathway in breast cancer which interacts with hypoxia in a bi-directional manner. Hypoxia increases the expression of Notch receptors and ligands in breast cancer cells, possibly via co-ordinated activity of HIFs and the drosophila mastermind homologue, mastermind-like protein 1, a transcriptional co-activator in Notch signalling. This is associated with increased expression of *slug* and *snail* genes while decreasing *E*-*cadherin* in a Notch pathway-dependent manner to increase cell invasiveness via EMT [88] reminiscent of that seen in MM. Thus, hypoxia acts via Notch signalling to trigger EMT via the *Snail*-*1* promoter and to induce cell invasion. Notch increases Snail-1 via two mechanisms; firstly, it has a direct interaction with the *Snail*-*1* promoter; secondly there is an intriguing synergistic mechanism whereby Notch up-regulated expression of the enzyme lysyl oxidase (LOX), which stabilises the Snail-1 protein once translated [89].

## The Emerging Role of Lysyl Oxidase in Bone Metastasis

Although lysyl oxidase (LOX) is most well known for being a collagen cross-linking enzyme, it has more recently been shown that LOX is an important factor in solid tumour progression. Tumour-derived LOX expression is driven by HIF1-α within hypoxic regions in tumours. LOX drives the motility of the invasive human ER^−^ Hs578T and human MDA-MB-231 breast cancer cell lines. The LOX inhibitor β-aminopropionitrile (BAPN) decreases cell motility in vitro, while focal adhesion kinase (FAK) and Src activity were positively correlated with LOX. In support of this, FAK and Src activation were shown to be decreased in a dose-dependent manner by the small molecule inhibitor of LOX; BAPN, and by catalase which degrades the LOX by-product; hydrogen peroxide [90]. We previously showed that patients with high LOX expressing ER^−^ tumours exhibit significantly worse distant metastasis-free survival and poorer overall survival than low LOX expressing ER^−^ counterparts. siRNA knockdown of LOX or inhibition with BAPN in human MDA-MB-231 breast cancer cells reduced hypoxia-driven invasion in nude mice, demonstrating a clear role for LOX in hypoxia-driven metastasis [91]. Consistent with these findings, BAPN injected into mice together with MDA-MB-231 human breast adenocarcinoma cells reduced number of metastases and preserved bone volume from osteolytic lesions [92].

Recently, we have added to our understanding of the seed and soil hypothesis in breast cancer to bone metastasis. We demonstrated that LOX secreted by a distal mammary tumours triggered osteolytic bone lesion formation. In this landmark study, LOX secreting 4T1 murine mammary carcinoma cells injected into the mammary fat pad of syngeneic BALB/c mice triggered bone lesion formation prior to invasion by cells from the primary tumour and could be abrogated by siRNA knockdown of LOX in the primary tumour cells. Injection of mice with conditioned media from 4T1 cells in a cell-free tumour-simulation model also drove lesion formation in bone in the absence of any primary tumour. This demonstrated that the secretome of the ER^−^ breast cancer cells was driving an imbalance in bone homeostasis in favour of osteolytic degradation. A lack of lesion formation in mice injected with conditioned media from 4T1 cells with shRNA knockdown for LOX confirmed a role for the enzyme. Histological analysis of femora from mice carrying 4T1 LOX secreting tumours revealed a reduction in osteoblast numbers concomitantly with an increase in osteoclast numbers, an effect that was abrogated by shRNA LOX knockdown in the primary tumour. Under the established model of osteoclast generation, both MCSF and RANKL cytokines are required for monocyte cell fusion and generation of functional osteoclasts [93]. However, culturing of enriched human CD14 + monocytes in the absence of RANKL but in the presence of LOX led to osteoclast formation via increased translocation to the nucleus of NFATc1, the master regulator of osteoclastogenesis. This effect was abrogated by using a LOX function-blocking antibody (targeting the active site of the enzyme), and by a dose-dependent treatment with catalase to remove LOX-generated hydrogen peroxide. Meanwhile, LOX activity reduced the proliferation of both primary murine calvarial osteoblasts and the human osteoblast like SaOS-2 line pushing these cells toward a more terminally differentiated phenotype with increased mineralisation. The in vivo effects of tumour secreted LOX on pre-metastatic bone lesion formation were preventable if mice were simultaneously treated with the potent bisphosphonate Zoledronic acid to prevent bone lesion formation [94]. This study opened up the possibility that bisphosphonate treatment can be used to help prevent metastasis of breast cancer to bone. At the same time, a large meta-study from the Early Breast Cancer Trialists’ Collaborative Group (EBCTCG) found that in post-menopausal women with breast cancer, the adjuvant treatment with bisphosphonates to block osteoporosis-driven bone remodelling led to a significant decrease in metastatic bone burden [95]. Together, these data suggest that targeting the remodelling of the bone microenvironment offers a viable therapeutic approach to blocking breast cancer to bone metastasis.

In contrast, studies by Tsukasaki et al. and Reynaud et al. observed an absence of osteoclast formation in the presence of LOX and in RANKL knockout models [96, 97]. However, the apparent contradictory nature of these findings may be explained by differences in experimental approach. In particular, Tsukasaki et al. used a truncated form of LOX that lacks the pro-peptide and part of the mature protein sequence, which together with larger N-terminal and His6 tags used for purification, could leave the enzyme without catalytic activity [98]. Studies by Reynaud et al., whilst supporting the overall findings of hypoxia-induced LOX expression in metastatic dissemination to the bone, they did not specify the exact form of LOX used [97]. It is also worth noting that unlike Cox et al. where human CD14 + monocytes were selected isolated and purified from human peripheral blood [94], Reynaud et al. also used a heterogeneous mixture of murine bone marrow cells [97] where the combined response to LOX remains to be fully understood. Tsukasaki et al. and Reynaud et al. did however find that LOX activity strongly synergises with, and amplifies RANKL action to significantly enhance osteoclastogenesis [96, 97], and Reynaud et al. also found that tumour-secreted LOX generates bone lesions consistent with findings by Cox et al. [94, 97]. Regardless, there is a clear need for further research into the precise molecular mechanisms underlying LOX-driven osteoclastogenesis.

## Summary and Future Directions

This review has covered some of the multitude of mechanisms that underlie unfavourable outcomes in hypoxia-driven aspects of cancer-associated bone disease. For primary bone cancers, survival rates have increased decade on decade as diagnosis methods and treatments have improved [6]. New hypoxia targeting drugs offer hope of increasing survival rates further. The best outcomes for patients with other cancers will be connected to prevention of metastasis to bone, which will come from new understandings about the underlying early mechanisms as exemplified by our own work. For those patients who are diagnosed after this transition step has occurred, the outlook remains one of managed decline, as the cancer is transiently held back with surgery, radiotherapy, and combination therapies. This significant challenge currently lies unresolved, yet perhaps one day secondary metastatic bone cancers will not just be manageable or treatable but curable too.
